# Deterioration of alveolar development in mice with both HIF-3α knockout and HIF-2α knockdown

**DOI:** 10.1186/s13104-018-3563-7

**Published:** 2018-07-09

**Authors:** Firman Zulkifli Amin, Toshiharu Yamashita, Osamu Ohneda

**Affiliations:** Department of Regenerative Medicine and Stem Cell Biology, Graduate School of Comprehensive Human Sciences, University of Tsukuba, 1-1-1 Tennodai, Tsukuba, 305-8575 Japan

**Keywords:** HIF-3α, HIF-2α, Double-mutant mice, Lung, Alveolar

## Abstract

**Objective:**

Earlier studies from our group using hypoxia-inducible factor 3α knockout mice showed impairments in lung remodeling and lung endothelial cells. Another research from our group demonstrated that impaired expression of hypoxia-inducible factor 2α induced compensatory expression of hypoxia-inducible factor 1α in hypoxia-inducible factor 2α knockdown mice. The present study uncovers more insights by extending the investigation, utilizing mice with both hypoxia-inducible factor 3α knockout and hypoxia-inducible factor 2α knockdown.

**Results:**

No mice with both hypoxia-inducible factor 3α knockout and hypoxia-inducible factor 2α knockdown died immediately after birth. The mice with both hypoxia-inducible factor 3α knockout and hypoxia-inducible factor 2α knockdown exhibited impaired alveolar sacs and lung alveolar structure and decreased endothelial cell numbers. Analysis of relative mRNA expression revealed depressed expressions of hypoxia-inducible factor 1α, vascular cell adhesion molecule 1, vascular endothelial cadherin, angiopoietin 2, Tie-2, and vascular endothelial growth factor in the lungs of mice with both hypoxia-inducible factor 3α knockout and hypoxia-inducible factor 2α knockdown compared to that in wild-type mice. Further analysis is needed to elucidate the impaired development occurred in the lung endothelial cells.

## Introduction

Hypoxia-inducible factors (HIFs) are heterodimers that consist of three oxygen-sensitive α-subunits (HIF-1α, HIF-2α, and HIF-3α) and a β-subunit, the aryl hydrocarbon receptor nuclear translocator (ARNT). HIFs act as regulators of the molecular hypoxic response [1, 2]; in a study examining normal alveolarization in fostered newborn rats, HIFs promoted alveolar development and regeneration by preventing and repairing oxygen-induced alveolar damage [3]. Nonetheless, HIF-1α inhibition using antisense knockdown in vitro during early lung development decreased vascular development and epithelial branching morphogenesis in lung explants [4]. In contrast, the conditional overexpression of HIF-1α in embryonic lung epithelium also impaired branching morphogenesis and lung maturation and affected vascular lung abnormalities, including hemorrhages and increased lymphangiogenesis [5]. Collectively, these data suggest that interference in the alveolar epithelium by oxygen pressure changes, including hypoxia, can affect alveolar homeostasis, leading to epithelial injuries and diseases such as lung fibrosis [6–9]. A previous study from our group that used HIF-3α knockout (−/−) mice showed impaired lung remodeling exhibited by the walls of the secondary septa in subdivided alveoli, and immunostaining of alveolar endothelial cells presented an increase in defective space in the interalveolar septa and hyperplasia of endothelial cells during the maturation of alveolar formation in these knockout mice [10]. Additionally, another study from our group revealed that these HIF-3α −/− mice showed impairments in lung endothelial cells presented by slow growth and a decreased number of tubes formed by endothelial cells [11]. Furthermore, a different but related study from our group demonstrated that impaired expression of HIF-2α in HIF-2α knockdown (kd/kd) mice induced compensatory expression of HIF-1α [12]. The present study uncovers more insights by extending the investigation from those previously stated findings of our group utilizing HIF-3α −/− and HIF-2α kd/kd (double-mutant) mice. In this study, male and female HIF-3α −/− and HIF-2α knockdown heterozygote (kd/+) mice were interbred, resulting in the double-mutant mice previously mentioned.

## Main text

### Materials and methods

#### Mice

All of the experiments performed were approved by the ethics committee of the University of Tsukuba. All wild-type (WT) and mutant mouse lines were of the C57BL/6J genetic background. HIF-2α kd/kd mice were generated as previously reported [13]. HIF-3α −/− mice were obtained as previously published [11]. Mating of 12 pairs of HIF-3α −/− and HIF-2α kd/+ mice for a breeding period of 1 year generated the double-mutant mice. The genome DNA was extract from tail of neonatal pup. The genotype of mouse was determined by polymerase chain reaction (PCR) as described previously [11, 13].

#### Isolation and culture of cells

The WT, HIF-2α kd/kd, HIF-3α −/−, and double-mutant mice were sacrificed by given the overdose treatment of anesthetic reagent (isoflurane; WAKO, Japan), and lung tissue from these mice were harvested as previously mentioned [14]. Lungs from WT, HIF-2α kd/kd, HIF-3α −/−, and double-mutant mice were dissected at postnatal day (P) 0 for hematoxylin and eosin staining and at postnatal week 6 for cell culture. Collagenase digestion (Nitta Gelatin, Osaka, Japan) was used to prepare the lung cell suspensions. Afterward, these cells were cultured in high glucose Dulbecco’s modified Eagle’s medium (DMEM; GIBCO) supplemented with 10% fetal bovine serum (FBS), 0.1 mmol/L nonessential amino acids, 2 mmol/L l-glutamine, penicillin–streptomycin, and 10^−4^ mol/L β-mercaptoethanol (HAVA medium) [15] and maintained without any addition of growth supplements.

#### Immunohistochemistry and section staining

The lung tissue samples from WT, HIF-3α −/−, and double-mutant mice were fixed with 4% paraformaldehyde combined with phosphate-buffered saline at 4 °C overnight and embedded in OCT compound (Sakura Finetek, Tokyo, Japan). Sections (5 μm) were then prepared for immunohistochemical and hematoxylin and eosin staining. Serial cryostat sections were incubated with CD31 (1:1000; clone: MEC 13.3; BD Biosciences, San Diego, CA, USA) antibody. The sections, after being washed, were incubated with an HRP-conjugated secondary antibody (1:2000; Vector Laboratories, Burlingame, CA, USA). Different sections were incubated with anti-HIF-1α, anti-HIF-2α, anti-vascular cell adhesion molecule 1 (VCAM-1), and anti-vascular endothelial cadherin (VE-cadherin) antibodies and stained using MOM™ kit (Vector Laboratories) referring to the manufacturer’s instructions.

#### Quantitative reverse transcription polymerase chain reaction (qPCR)

Total RNA was obtained from samples (*n* = 3) with the use of the extraction reagent (Sepasol-RNA I Super G; Nakalai Tesque, Kyoto, Japan). cDNA was then synthesized by reverse transcription (ReveTra Ace; TOYOBO, Osaka, Japan). The expression level was analyzed by using the 7500 Fast Real-Time PCR machine (Applied Biosystems, Carlsbad, CA, USA) with SYBR-green (Life Technologies, Carlsbad, CA, USA). Experiments were performed in triplicate, and the resulting data were analyzed by the delta CT method.

### Results

#### No double-mutant mice died immediately after birth

Interbreeding between male and female HIF-3α −/− and HIF-2α kd/+ mice resulted in 198 offspring within 1 week. Of the total number of offspring, 5.1% were surprisingly double-mutant mice, with very few pups still alive at birth, 34.3% were HIF-3α −/− and HIF-2α +/+, and 60.6% were HIF-3α −/− and HIF-2α kd/+. Almost none of the double-mutant mice were alive at birth.

#### The double-mutant mice had impaired alveolar sacs and lung alveolar structure and decreased endothelial cell numbers

**Fig. 1:**
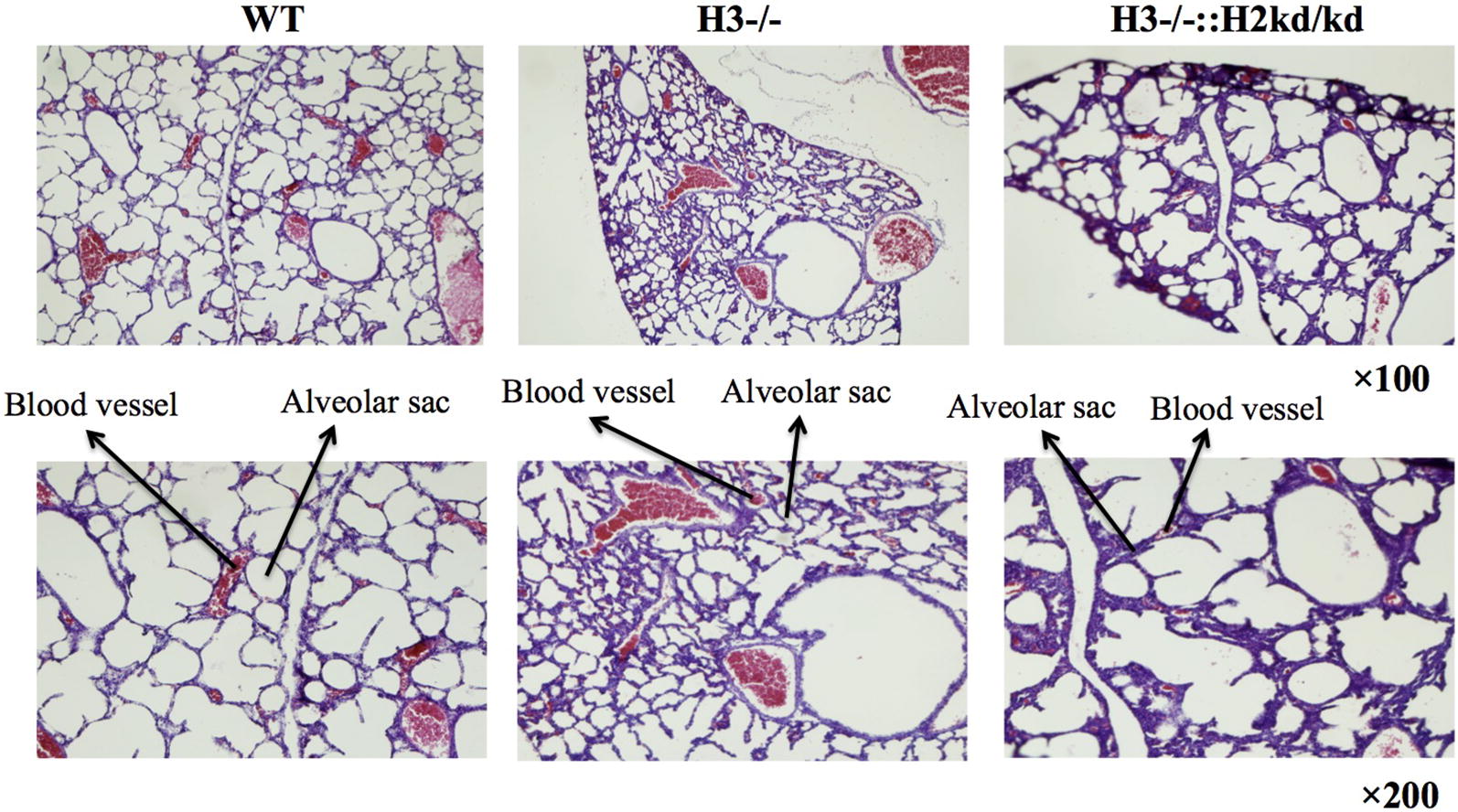
The double-mutant mice had impaired alveolar sacs. The lung of WT mice, HIF-3α −/− mice, and double-mutant mice at P0 was examined based on hematoxylin and eosin staining. H3, hypoxia-inducible factor 3α; H2, hypoxia-inducible factor 2α; ::, intercrossed with; −/−, knockout; kd/kd, knockdown; WT, wild-type

**Fig. 2:**
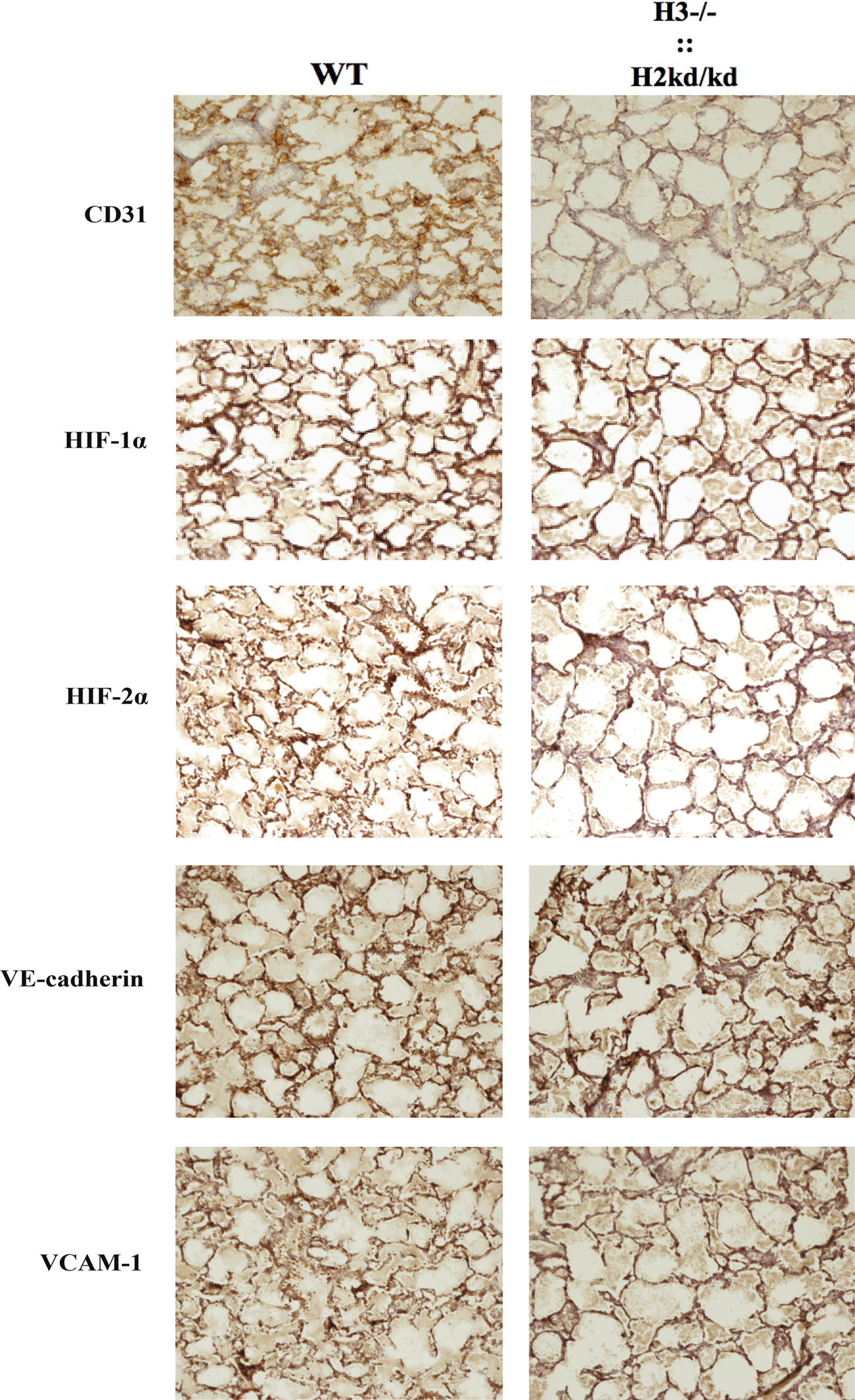
The double-mutant mice had impaired lung alveolar structure and decreased endothelial cell numbers. Immunohistochemistry analysis performed to sections from neonatal WT and double-mutant mice at 2–3 days of age using CD31, HIF-1α, HIF-2α, VE-cadherin, and VCAM-1 staining. HIF-1α, hypoxia-inducible factor 1α; HIF-2α, hypoxia-inducible factor 2α; H3, hypoxia-inducible factor 3α; H2, hypoxia-inducible factor 2α; ::, intercrossed with; −/−, knockout; kd/kd, knockdown; VE-cadherin, vascular endothelial cadherin; VCAM-1, vascular cell adhesion molecule 1; WT, wild-type

Hematoxylin and eosin staining of the lung in WT, HIF-3α −/−, and double-mutant mice was performed at P0 (Fig. 1). We found that alveolar sacs were almost imperceptible in the lungs of double-mutant mice, and the appearance of the blood vessels in the lungs of these mice was different than that in the lungs of HIF-3α −/− and WT mice. There is no phenotype data for HIF-2α kd/kd mice at present. Moreover, immunohistochemistry analysis of sections from neonatal WT and double-mutant mice at 2–3 days of age (Fig. 2) by CD31 staining showed impaired lung vessel structure of the neonatal double-mutant mice accompanied with the decreased of endothelial cell numbers. HIF-1α and HIF-2α staining of the neonatal double-mutant mice revealed the decreased expressions of both HIFs and endothelial cell numbers. VCAM-1 and VE-cadherin staining of the neonatal double-mutant mice showed their expressions and also the decreased of endothelial cell numbers.

#### The double-mutant mouse lung exhibited depressed expressions of HIF-1α, VCAM-1, VE-cadherin, Ang-2, Tie-2, and VEGF

**Fig. 3:**
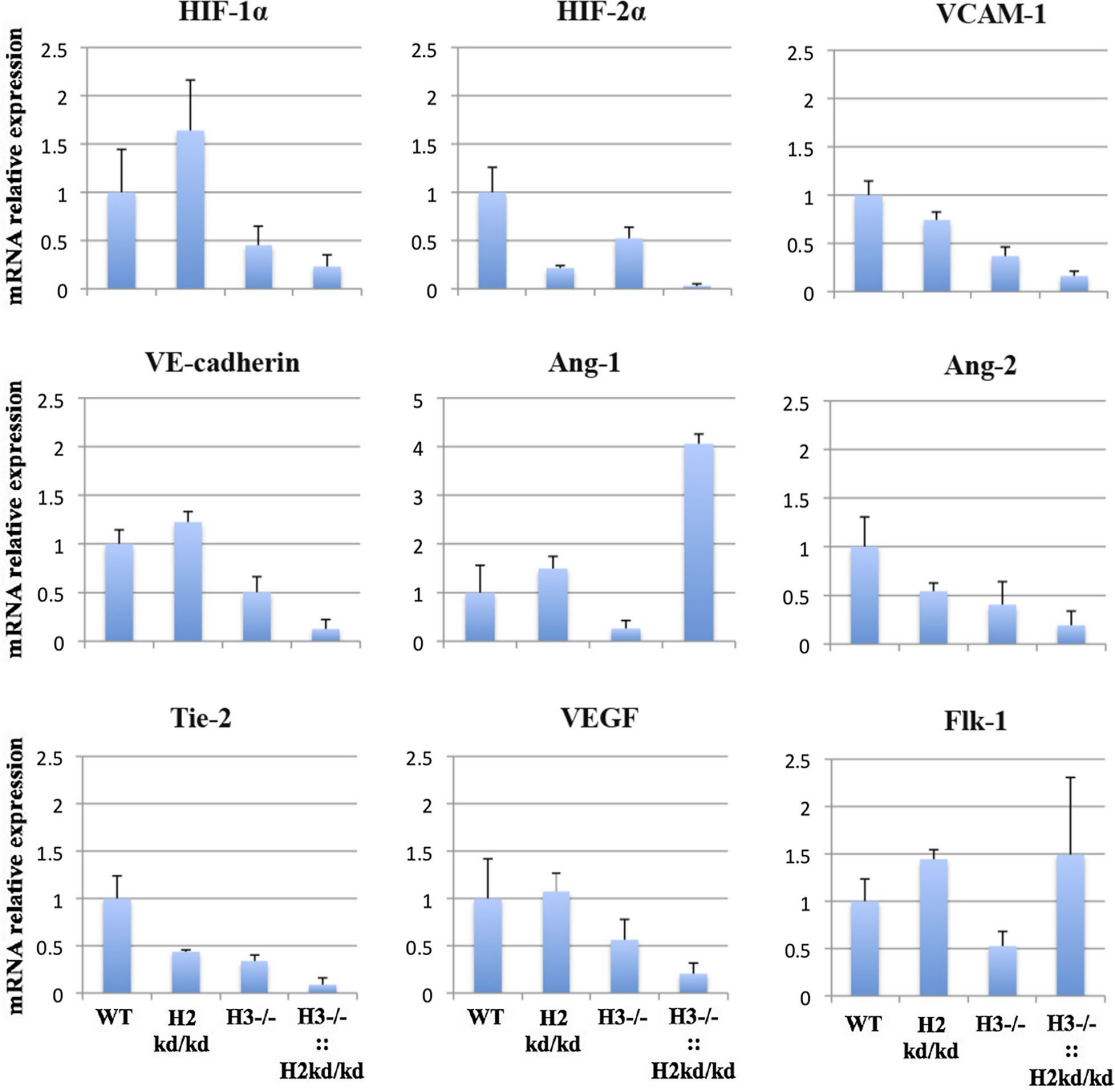
The double-mutant mouse lung exhibited depressed expressions of HIF-1α, VCAM-1, VE-cadherin, Ang-2, Tie-2, and VEGF. The utilizing qPCR performed the analysis of mRNA relative expressions for HIF-1α, HIF-2α, VCAM-1, VE-cadherin, Ang-1, Ang-2, Tie-2, VGEF, and Flk-1 that normalized to β-actin. Ang-1, angiopoietin 1; Ang-2, angiopoietin 2; H3, hypoxia-inducible factor 3α; H2, hypoxia-inducible factor 2α; ::, intercrossed with; −/−, knockout; kd/kd, knockdown; HIF-1α, hypoxia-inducible factor 1α; HIF-2α, hypoxia-inducible factor 2α; mRNA, messenger ribonucleic acid; VCAM-1, vascular cell adhesion molecule 1; VE-cadherin, vascular endothelial cadherin; VEGF, vascular endothelial growth factor; WT, wild-type

We analyzed the relative mRNA expression levels of potentially related genes HIF-1α, HIF-2α, VCAM-1, VE-cadherin, angiopoietin 1 (Ang-1) and 2 (Ang-2), Tie-2, vascular endothelial growth factor (VEGF), and Flk-1. The analysis utilized the whole lung tissue from neonatal WT, HIF-2α kd/kd, HIF-3α −/−, and double-mutant mice at 2–3 days of age (Fig. 3). Surprisingly, we found that HIF-1α, VCAM-1, VE-cadherin, Ang-2, Tie-2, and VEGF expressions were reduced in the lung of the neonatal HIF-3α −/− mice and even more depressed in the lung of the neonatal double-mutant mice. We also found that HIF-2α and Flk-1 expressions were similarly reduced in the lung of the neonatal HIF-3α −/− mice. Additionally, Ang-1 was unexpectedly expressed excessively in the lung of the neonatal double-mutant mice.

### Discussion

The present study has elucidated that no double-mutant mice died immediately after birth. This new finding is slightly opposed to the common knowledge that HIF deficiency is immediately postnatally lethal. The reason that none of the double-mutant mice died postnatally remains unclear. Furthermore, this study is the first report that the alveolar sacs of the double-mutant mice are impaired. The current result has broadened the understanding from our previous study that revealed incomplete alveolar spaces in HIF-3α −/− mice [10]. Immunohistochemistry results have shown that the decreased of endothelial cell numbers, which impair proliferative and angiogenic activities appeared to contribute to impaired lung alveolar structure of the neonatal double-mutant mice. In addition, the lung endothelial cells isolated from the neonatal double-mutant mice showed the impaired proliferative ability (data not shown), suggesting the functional impairment of these cells by both HIF-3α −/− and HIF-2α kd/kd. The decreased of HIF-1α and HIF-2α expressions causing of lacking to overcome the happening of oxygen homeostasis disruption also deteriorate such lung alveolar structure. Such pathological features may provide further insight into the molecular mechanism of alveolar development especially for further investigation at the embryonic stage. We next acknowledged that our current results on the gene expressions in the lung of the neonatal HIF-3α −/− mice are partly in line as well as in contrary to the results showed on our former study utilizing the adult HIF-3α −/− mice [11]. The contrary results in the current study are mostly correlated to the angiogenic gene regulations due to the depressed mRNA levels of HIF-1α, HIF-2α, VCAM-1, VE-cadherin, Ang-2, Tie-2, HIF-2α, VEGF, and Flk-1 in the lung of the neonatal HIF-3α −/− mice. The mRNA levels of HIF-1α, VCAM-1, VE-cadherin, Ang-2, Tie-2, and VEGF are even more depressed in the lung of the neonatal double-mutant mice. Our understanding of these new findings may be further understood in light of knowledge that the VEGF/Flk-1 and angiopoietin/Tie-2 signaling pathways are vital for the maintenance of endothelial cell homeostasis [16–18], which then can explain impaired alveolar sacs and lung alveolar structure conditions. Ang-1 that is interestingly highly expressed even though HIF-1α is inversely expressed in the lung of the neonatal double-mutant mice, which against the previous report concluding HIF-1α explicitly targets Ang-1 [19] may indicate other regulations involved. Overall, these findings should be investigated further to firmly elucidate the impaired development occurred in the lung endothelial cells.

## Limitations

The limited number of double-mutant mice that survive postnatally may hinder the acquisition of sufficient samples for the isolation and identification of the lung endothelial cells.

## Abbreviations

ATII: alveolar type 2
Ang-1: angiopoietin 1
Ang-2: angiopoietin 2
ARNT: aryl hydrocarbon receptor nuclear translocator
cDNA: complementary deoxyribonucleic acid
DMEM: Dulbecco’s modified Eagle’s medium
FBS: fetal bovine serum
HAVA: high glucose Dulbecco’s modified Eagle’s medium supplemented with 10% fetal bovine serum, 0.1 mmol/L nonessential amino acids, 2 mmol/L l-glutamine, penicillin–streptomycin, and 10^−4^ mol/L β-mercaptoethanol
HIF: hypoxia-inducible factors
HIF-1α: hypoxia-inducible factor 1α
HIF-2α: hypoxia-inducible factor 2α
HIF-3α: hypoxia-inducible factor 3α
kd/kd: knockdown
kd/+: knockdown heterozygotes
mRNA: messenger ribonucleic acid
*n*: sample number
OCT: optimum cutting temperature
P: postnatal day
PCR: polymerase chain reaction
qPCR: quantitative real-time polymerase chain reaction
RNA: ribonucleic acid
VCAM-1: vascular cell adhesion molecule 1
VE-cadherin: vascular endothelial cadherin
VEGF: vascular endothelial growth factor
WT: wild-type
−/−: knockout
+/+: homozygotes
